# Alcohol Hangover Differentially Modulates the Processing of Relevant and Irrelevant Information

**DOI:** 10.3390/jcm9030778

**Published:** 2020-03-12

**Authors:** Antje Opitz, Christian Beste, Ann-Kathrin Stock

**Affiliations:** Cognitive Neurophysiology, Department of Child and Adolescent Psychiatry, Faculty of Medicine, TU Dresden, Fetscherstr. 74, 01307 Dresden, Germany; christian.beste@ukdd.de (C.B.); ann-kathrin.stock@ukdd.de (A.-K.S.)

**Keywords:** alcohol, hangover, distraction, stimulus-response binding, distractor-response binding, theory of event coding

## Abstract

Elevated distractibility is one of the major contributors to alcohol hangover-induced behavioral deficits. Yet, the basic mechanisms driving increased distractibility during hangovers are still not very well understood. Aside from impairments in attention and psychomotor functions, changes in stimulus-response bindings may also increase responding to distracting information, as suggested by the theory of event coding (TEC). Yet, this has never been investigated in the context of alcohol hangover. Therefore, we investigated whether alcohol hangover has different effects on target-response bindings and distractor-response bindings using a task that allows to differentiate these two phenomena. A total of *n* = 35 healthy males aged 19 to 28 were tested once sober and once hungover after being intoxicated in a standardized experimental drinking setting the night before (2.64 gr of alcohol per estimated liter of body water). We found that alcohol hangover reduced distractor-response bindings, while no such impairment was found for target-response bindings, which appeared to be unaffected. Our findings imply that the processing of distracting information is most likely not increased, but in fact decreased by hangover. This suggests that increased distractibility during alcohol hangover is most likely not caused by modulations in distractor-response bindings.

## 1. Introduction

Driving under the influence of alcohol is prohibited and legally sanctioned in most countries, as alcohol drastically impairs the required cognitive and motor skills, thus putting the driving individual as well as co-drivers and bystanders at substantial risk of accidents and injury [1,2,3,4,5]. Even after the acute intoxication has worn off and blood alcohol levels (BAC) have returned to 0.00‰, cognitive, attentional, and psychomotor functions likely remain impaired to a certain degree [6,7,8,9,10,11]. While there is currently no neurobiochemical marker that could help to reliably detect the presence of alcohol hangover [12], being hungover may not only be disastrous for driving a vehicle, but also for various daily activities including safety-sensitive tasks at work and at home. Surprisingly, the mechanisms underlying such hangover-associated behavioral deficits are still not very well understood, as compared to the effects of acute intoxication.

**Figure 1:**
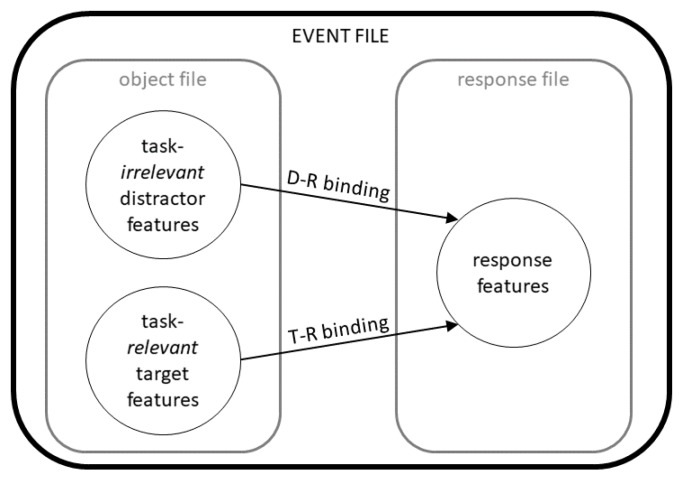
Schematic illustrations of the bindings postulated by the theory of event coding (TEC). Abbreviations: D-R = distractor-response; T-R = target-response.

Alcohol hangover is commonly defined as a “combination of mental and physical symptoms, experienced the day after a single episode of heavy drinking, starting when BAC approaches zero” [13]. The findings on cognitive and behavioral effects of alcohol-induced hangover in different functional domains are not always consistent (for review, please see Mackus et al. [11]), but there is consensus that attentional deficits are one of the factors that likely underlie hangover-associated driving impairments [7,11,14]. Another important factor for impaired driving skills are slowing and impairments of motor responses and coordination during hangover [6,7,11,14,15]. Even though distraction is recognized as a major risk factor for impaired driving [16], we still know rather little about whether and how alcohol hangover changes the way we process and respond to distracting information, other than that hangover may influence response selection and conflict monitoring, thus impairing goal-directed behavior [17,18,19]. Specifically, only very little is known about whether alcohol hangover differently modulates the integration and processing of irrelevant vs. relevant information, even though this may give rise to conflicts and distraction at later information processing stages. Against this background, obtaining a better understanding of hangover-induced changes in distractor processing is important. From a mechanistic point of view, driving performance, as well as some forms of occupational performance (for review, please see Mackus et al. [11]), may not only be compromised by hangover-induced impairments in attention and psychomotor functions, but also by the phenomenon of stimulus-response (S-R) binding. In this context, the theory of event coding (TEC) [20] has provided compelling evidence for why and how we respond to distracting information (Figure 1). While it has not been developed to explain alcohol effects per se, it is a widely known theory which may be used to elucidate mechanisms potentially underlying hangover-induced changes. In short, it states that sensory information on all kinds of different stimuli will be “bound” (i.e., associated) in a so-called object file, while all response-related information is bound/associated in the so-called “response file”. Additionally, stimulus and response features become associated with each other in the “event file”, thus constituting the S-R link [21]. Importantly, the event file binds stimulus and response features irrespective of whether a given stimulus feature/sensory information is functionally relevant for the response we intend or need to carry out [21]. Instead, temporal co-occurrence is the main determinant of whether sensory input becomes associated with a given response [21]. As a result, any distractor that will occur at the same time as a response-relevant (target) stimulus feature, will also find entry into the event file. Following a pattern-completion logic, the entire event file, including the previously associated response, will become activated once any part of a pre-existing event file is re-encountered [20]. Importantly, this also holds true for distractor information so that task-irrelevant information may lead to a similar reactivation of an event file as task-relevant information [20]. This way, a distractor, especially if previously encountered, may prime certain responses - even when they are no longer needed or correct.

While previous studies have suggested that acute alcohol intoxication does not seem to strongly impair S-R binding [22], this has never been investigated in the hungover state, which may functionally differ from the intoxicated state [17,18]. Of note, there are several mechanisms which could drive such differences, including differential modulation of *gamma-aminobutyric acid* (GABAergic) signaling by ethanol and its metabolite acetaldehyde, which is likely responsible for feeling hungover [23,24,25], and immunological parameters, including those related to inflammation [26,27]. To the best of our knowledge, it has furthermore never been investigated whether potential alcohol effects on this phenomenon could be different for the S-R binding of response-relevant target information vs. that of response-irrelevant distractor information. Stock et al. [22] investigated acute intoxication effects using a paradigm, which does not separately account for target- and distractor-response binding. We therefore chose a paradigm that allows to make this dissociation in order to investigate our research question in detail. Since the sensory processing of stimulus information might be altered during hangover due to increased sensory information processing speed [17], it could be conceivable that S-R binding, including distractor-response binding, is in fact enhanced during alcohol hangover. Such an enhancement could constitute a risk factor for distractibility and resulting safety issues for activities like driving (via the automatic reactivation of previously associated and thus potentially incorrect responses [28]).

To investigate this research question, we subjected *n* = 35 healthy young males to an experimental within-subject study design that was comparable to the procedure used in previously published hangover studies from our group [18,19]. Each participant was once tested sober and once after having consumed 2.64 gr of alcohol per estimated liter of body water on the night before testing. A paradigm introduced by Moeller et al. [29] was used to separately investigate the effects of target-response and distractor-response binding. In short, the paradigm combines target and distractor stimuli in a shared visual array that requires different manual responses (button presses) from the participants. By systematically and independently varying the repetition vs. change of target information, distractor information, and required response, the task allows to dissociate the different binding effects: The effect of distractor-response (D-R) binding should become evident in the statistical main effects of the distractor (i.e., be reflected by the effect size of distractor repetition vs. distractor change). Likewise, the effects of target-response (T-R) binding should become evident in statistical main effects of the target (i.e., be reflected by the effect size of whether the target and/or required response changed). Lastly, distractor-response binding may also show in the statistical interaction of distractor and response effects, as the automatic response tendency induced by the distractor should vary across response repetition vs. response change [30]: Distractor repetition should be beneficial whenever the repetition of a given response is required because the repetition of the distractor then reactivates the correct response. Opposing this, distractor repetition should be detrimental whenever a given response needs to be changed because the repetition of the distractor then reactivates an incorrect response.

In summary, we investigated whether S-R binding is altered during alcohol hangover against the background of potentially different sensory processing of distracting stimulus information [17,18]. We further investigated whether there are potentially different hangover effects on the binding of task-/response-relevant vs. irrelevant information, as this might explain why and how distractibility may be increased during alcohol hangover.

## 2. Experimental Section

### 2.1. Participants

The original study sample comprised *n* = 37 healthy males aged between 19 and 28 years, who were recruited from the local university (TU Dresden) and local nightlife district. Inclusion criteria were right-handedness, normal or corrected-to-normal vision, absence of color blindness, no acute or chronic somatic psychological, or neurological illnesses, and no intake of medication affecting kidney or liver function, or the central nervous system. Participants’ drinking habits were assessed during a telephone screening using the alcohol use disorders identification test (AUDIT) [31,32]. An AUDIT score below 16 specifies low-to-moderate risk of alcohol use disorder (AUD) [31]. Almost all participants scored below this cut off and therefore reported no drinking habits at high-risk. In total, *n* = 4 participants scored between 16 and 19 indicating harmful use of alcohol [31] but did not meet the criteria for the diagnosis of an AUD according to the International Classification of Diseases (ICD-10). None of the participants scored above 19, which would have indicated a high risk/presence of an AUD [31]. Although harmful drinking may potentially have detrimental effects on cognitive functions [33], previous findings from our lab suggested that young frequent binge drinkers (who engaged in equal, and/or more binge-drinking than our current sample) seem to have no significant behavioral impairments in executive functioning [34].

Exclusion criteria used to minimize the number of individuals who might not cope well with the amount of experimentally administered alcohol were an overall AUDIT score below 2 points, less than 13 self-reported binge-drinking nights over the past 12 months, and not being markedly drunk at least once in the past 12 months. Exclusion criteria used to minimize the number of individuals with high alcohol tolerance and/or high risk of AUD were an overall AUDIT score above 19 points [31], and self-reported (nearly) daily occurrences of either binge drinking behavior, and/or alcohol-induced memory problems, and/or failure to fulfill daily routine tasks due to alcohol consumption. All participants provided written informed consent and received a reimbursement of 80€. The study was conducted according to the Declaration of Helsinki. Ethical approval was given by the ethics committee of the Faculty of Medicine of the TU Dresden, Germany (EK293082014). Eventually, *n* = 35 participants (23.1 ± 2.7 years old) were included in the statistical analyses because one participant was excluded due to performance accuracy close to chance level (i.e., below 55%) in at least one experimental task condition and another participant was excluded because of his high residual breath alcohol concentration (BrAC = 0.45‰) at the hangover session, which would have taken a waiting time of 4 to 5 additional hours until sobriety and he was not able to invest the required time on that day. Please also note that the sample in this publication largely overlapped with that of a previous publication investigating the influence of alcohol hangover on meta-control/the interplay between controlled and automated processes [19].

### 2.2. Experimental Design

**Figure 2:**
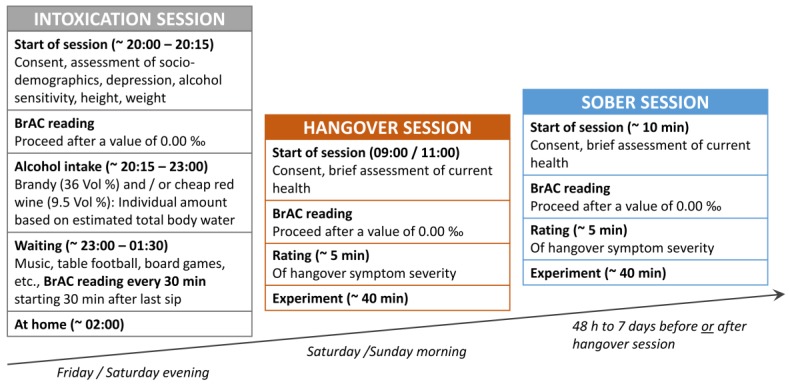
Outline of the intoxication, hangover, and sober sessions. Each participant was tested twice, once sober and once hungover, with a delay of a min. 48 h to max. 7 days between both sessions. The order of both sessions was counterbalanced across all participants. Written informed consent and a brief evaluation of the current health status was provided at the start of each session. Rating of hangover symptoms and recording of task performance was only started in case of a breath alcohol concentration (BrAC) of zero on both sessions. To induce alcohol hangover symptoms, participants were experimentally intoxicated at our laboratory on a Friday or Saturday evening starting from 20:00 (8 pm). Participants provided their written consent and completed questionnaires on sociodemographic data, height, weight, alcohol sensitivity, and depression. BrAC was measured before they started drinking to ensure sobriety. An individually-measured alcohol amount was consumed from approximately 20:15 to 23:00. BrAC was measured 30, 60, 90, and 120 min after the last alcoholic drink was finished. At around 01:30, participants were brought home via cab, given that their BrAC values were already declining, they had no impairments of consciousness, and no major walking/coordination impairments. Participants came back to the laboratory in the next morning between 09:00 and 11:00 to complete their hangover session.

The experimental design was identical to that described in previous publications by our group [18,19], and is illustrated in Figure 2.

Each participant was tested twice (once sober and once hungover) with a delay of a minimum of 48 h to a maximum of 7 days between the sober and hangover sessions. The order of both sessions was counterbalanced across the sample. Participants were asked to neither use nicotine, nor caffeine or guarana within four hours before the start of each session. At the start of each session, BrAC was measured using the breathalyzer “Alcotest 3000” following the instructions by the manufacturer (Drägerwerk, Lübeck, Germany). The experimental procedure in the sober and hangover sessions only proceeded in case of a BrAC of 0.00‰. On the night before the hangover session, participants were experimentally intoxicated at our laboratory in order to induce hangover symptoms. For this, we asked groups of 4 to 8 participants to join a drinking session on a Friday or Saturday evening (starting at 20:00 and ending at approximately 01:30/02:00). The subsequent hangover session was planned for the following morning (that is either on Saturday or on Sunday, starting between 09:00 and 11:00) to slightly reduce sleep duration, as the amount of sleep time is negatively related to hangover severity [35].

### 2.3. Experimental Intoxication Procedure

The experimental intoxication procedure applied to cause hangover symptoms followed the same protocol as described in previous publications [18,19] and is briefly outlined in Figure 2. By using a version of the equation by Widmark [36] and Watson et al. [37], an individual amount of alcohol was calculated for each participant at the beginning of the intoxication session. It estimates the total body water (TBW) in liters based on an individual’s sex, age, weight, and height and specifies the alcohol amount in grams which is required to be dissolved in the body water to attain a given intoxication level (2.64 gr of alcohol per estimated liter of body water). This limited the provided alcohol amount so that it was physiologically impossible to reach a BrAC of more than 2.0‰ (which would only have been possible to reach if all of the alcohol had been dissolved in TBW at once). Realistically, the amount of provided alcohol translates to a mean BrAC of 1.6‰ in case it is consumed at once and on an empty stomach (i.e., with an expected resorption deficit of about 20%). However, participants were encouraged to eat a full dinner before the intoxication session, as a full stomach typically increases the resorption deficit (approx. 30%–40%). Additionally, the experimenters ensured a minimum consumption duration of 2 h in order to prevent the participants from drinking the entire amount at once and potentially experiencing “overshooting” (i.e., a very rapid increase in blood alcohol levels that might initially lead to otherwise unexpectedly high blood alcohol concentrations and associated complications like vomiting). As a consequence of these precautions, the participants were expected to achieve a mean BrAC of approximately 1.2‰ with only a small chance of reaching BrAC values beyond 1.6‰. The equation used to calculate the individual amount of alcohol and the protocol used to record the alcohol consumption of each participant is available online at https://osf.io/ktgyr/. As alcoholic beverages with high congener content are more likely to induce a (more severe) hangover [7], we only offered cheap red wine (9.5 vol %) and/or cheap brandy (36 vol %). To keep the quantity and speed of alcohol consumption similar across beverages, the experimenters served standardized portions of 50 mL brandy (14 g alcohol) or 200 mL red wine (15 g alcohol). For each standardized portion of alcoholic beverage, participants could choose whether they wanted to drink it pure, chilled on ice, or mixed with orange lemonade, caffeine-free coke, or ginger ale. Participants were supplied with tap water and snacks (wine gums and chips), the use of which was not recorded. Participants were additionally allowed to smoke cigarettes while drinking, as this has been suggested to enhance hangover severity [38,39]. In total, *n* = 9 participants appreciated this offer, *n* = 8 of whom reported to smoke regularly. BrAC was assessed 30, 60, 90, and 120 min after the last alcoholic beverage was consumed.

### 2.4. Questionnaires

At both the sober and hangover session, participants rated the severity of their hangover symptoms. For this purpose, we used the 11-point Likert-scale by van Schrojenstein Lantman and colleagues [13]. This rating scale enlists 23 hangover symptoms, and rating options range from 0 points (no symptoms) to 10 points (extreme symptoms). We additionally asked participants to rate their sleep quality/sleep problems of the previous night in the same way. Furthermore, participants indicated the number of sleeping hours they had received the night prior to each testing session. Of note, they were explicitly asked to rate each symptom irrespective of whether or not they had been drinking the night before and of whether or not they attributed their symptoms to alcohol.

At the intoxication session, participants provided sociodemographic information and filled in Beck’s depression inventory (BDI) [40] to identify depressive symptoms, as depression could have potentially affected cognitive performance. Finally, the alcohol sensitivity questionnaire (ASQ) was used to assess susceptibility to known alcohol effects, like experiencing a hangover or feeling relaxed [41]. The ASQ further distinguishes between alcohol-related experiences associated with lower doses (ASQ_light_) and with heavier doses (ASQ_heavy_). While we ran detailed analyses on the role of alcohol sensitivity in a previous publication [19], we refrained from detailed alcohol sensitivity analyses in the current study, as this did not seem to contribute to hangover-associated behavioral changes in the previous study [19].

### 2.5. Task

**Figure 3:**
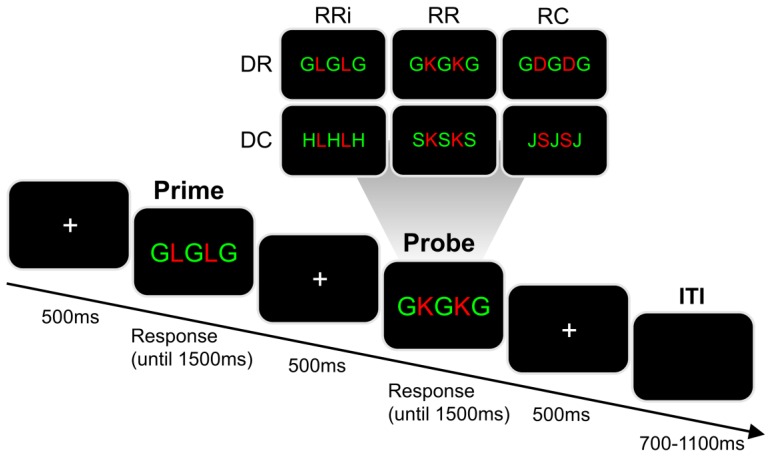
Illustration of the time course in milliseconds (ms) of a single trial in the distractor-response paradigm [29]. In each trial, a white fixation cross was centrally shown on a black screen for 500 ms. This was followed by a centrally presented prime array consisting of five letters. The prime array presentation was terminated either by a button press in response to the red target letters, or after 1500 ms (in case of a missing response). After another presentation of a central white fixation cross for 500 ms, a probe array was centrally presented. The probe array also consisted of five letters and its presentation was again terminated by a button press in response to the red target letters or ended after 1500 ms. The inter-trial interval (ITI) was jittered between 700 and 1100 ms. The upper half of the figure illustrates exemplary probe arrays for each combination of conditions in relation to the prime array used for illustration in this figure (“G L G L G”). Depending on whether the target and/or distractor stimulus were repeated or changed between prime and probe array, six different combinations of conditions were distinguished. In terms of the distractor stimuli, distractor repetition (DR) and distractor change (DC) were distinguished. In terms of the required response, response repetition when the target stimulus was identical (RRi), response repetition when the target stimulus differed (RR), and response change (RC) were distinguished.

In order to be able to dissociate the effects of alcohol hangover on the processing and binding of response-relevant (target) information and response-irrelevant (distractor) information, we used a paradigm introduced by Moeller et al. [29]. Figure 3 illustrates the paradigm.

The stimuli were presented on a 17′’ high quality flat screen monitor and keyboard responses were recorded using Presentation^®^ software (Version 16.5, Neurobehavioral Systems, Inc., Berkeley, CA, USA). The time course of a single trial was as follows: A white fixation cross was centrally presented on a black screen for 500 ms. This was followed by a centrally presented prime stimulus consisting of five horizontally aligned letters. The presentation of the prime array was either ended by a button press, or after 1500 ms had elapsed (in case of a missing response). Next, a central white fixation cross was presented for 500 ms. In case of an incorrect or missed response to the prime, a 500 ms error feedback was additionally displayed on the screen (i.e., the word “Fehler”, translating to “error”). Then, another five letters were centrally displayed as the probe array. Like for the prime array, the presentation of the probe array was either terminated by a button press, or after 1500 ms had elapsed (in case of a missing response). The probe array was also followed by the presentation of a central white fixation cross. In the case of an incorrect or missed response to the probe, an additional 500 ms error feedback was presented on the screen (i.e., the word “Fehler”, translating to “error”). The inter-trial interval (ITI) randomly varied between 700 and 1100 ms. The paradigm comprised a total of 576 trials, which were divided into six equally-sized blocks. Participants were offered to take breaks in-between these blocks.

With respect to S-R mapping, two out of eight letter stimuli (S, D, F, G, H, J, K, L) each formed a group. This resulted in four stimulus groups, each associated with a different response button on a standard QWERTZ keyboard. In detail, S and D required pressing the left Ctrl button with the left middle finger; F and G required pressing the left Alt button with the left index finger; H and J required pressing the right Win button with the right index finger; and K and L required pressing the right Ctrl button with the right middle finger. In each array, there were always two target letter stimuli which were shown in red font color. They were horizontally flanked by three distractor letter stimuli, which were shown in green font color. Please note that the target and distractor stimuli were never identical and always associated with different response buttons, even though only the targets required a single button press response. Participants were asked to respond to the red target letters while ignoring the green distractor letters. They were instructed to respond by pressing the appropriate button as quickly and as accurately as possible. Each trial asked for a first response to the prime array and a second response to the subsequent probe array.

The task allows to distinguish six combinations of conditions resulting from the modification of the distractor stimulus and/or response between prime and probe. All conditions are exemplarily illustrated in Figure 3. Regarding the distractor stimuli, there could be a distractor repetition (DR) or a distractor change (DC) between the prime and probe. Regarding the required response, there could be a repetition of the correct response button when the target stimulus was identical for both prime and probe (RRi), a repetition of the correct response button when the target stimulus differed between prime and probe (RR), and a change of the correct response button (RC). Each combination of conditions was presented equally often. In each of the conditions, all possible combinations of probe target and probe distractor were equally likely (considering that target and distractor stimuli could never require the same response). Prime and probe distractors were identical in all DR trials, while the prime distractor was randomly picked from one of the other response groups in all DC trials (e.g., when the probe distractor was the letter “G”, the prime distractor could not be the letter “G” or “F”). Additionally, the prime target was randomly picked from one of the other response groups in the RC condition. A total of 30 practice trials was performed before the experiment started. The task itself took around 40 min to finish.

### 2.6. Statistical Analyses

Statistical analyses included only trials in which participants responded correctly to both the prime and probe arrays. For each participant, trials with prime and/or probe response times (RTs) beyond ±2 standard deviations from the individual’s mean RT in the respective task condition were rejected in order to reduce outlier effects. The obtained correct probe RTs and probe accuracy were analyzed with SPSS Statistics 25 (IBM Corp. Released 2017. IBM SPSS Statistics for Windows, Version 25.0. Armonk, NY, USA: IBM Corp.) using separate repeated-measures ANOVAs. Status (hangover vs. sober), response (response repetition to identical target (RRi) vs. response repetition to other target (RR) vs. response change (RC)), and distractor (distractor repetition (DR) vs. distractor change (DC)) were used as within-subject factors. Greenhouse–Geisser correction was applied whenever necessary. Post hoc multiple comparisons were Bonferroni-corrected. All behavioral variables were analyzed for normal distribution indicated by Kolmogorov–Smirnov tests. As this assumption was not met for RTs in a few conditions and for most accuracy conditions, all significant main effects and post hoc tests were additionally tested for significance using non-parametric Wilcoxon signed-rank tests. All descriptive statistics provide the mean value and the standard error of the mean (SEM) as a measurement of variability. Descriptive and behavioral data, as well as all statistical analyses, are available online at https://osf.io/ktgyr/.

## 3. Results

### 3.1. Sample

Out of *n* = 35 participants, *n* = 17 had their hangover session before their sober session and *n* = 18 had their sober session before their hangover session. Sociodemographic characteristics, questionnaire scores, and alcohol-related data are detailed in Table 1. Subjective sleep and hangover ratings on both sessions are provided in Table 2. A total of *n* = 2 participants reported to have never experienced a hangover over their entire lifetime as assessed by the first ASQ item. Only one of them belonged to a total of *n* = 2 participants who indicated to not have experienced any overall hangover at the hangover session, meaning they rated the first item of the hangover severity rating by van Schrojenstein Lantman et al. [13] with 0 points. Nevertheless, both of these participants reported light hangover complaints for some of the symptoms in this rating, suggesting that none of them were entirely free of any hangover symptoms (please note that add-on analyses on hangover severity and symptoms can be found in the Supplementary Materials). Furthermore, a paired t-test showed that the number of standard drinks consumed at the intoxication session (16.74 drinks ± 0.25) was significantly larger than the maximal number of drinks participants indicated to be able to drink before experiencing a hangover (8.94 drinks ± 0.69; *t*_(32)_ = |10.696|; *p* < 0.001). Thus, the amount of alcohol administered in this study exceeded the self-reported hangover threshold.

### 3.2. Behavioral Data

**Figure 4:**
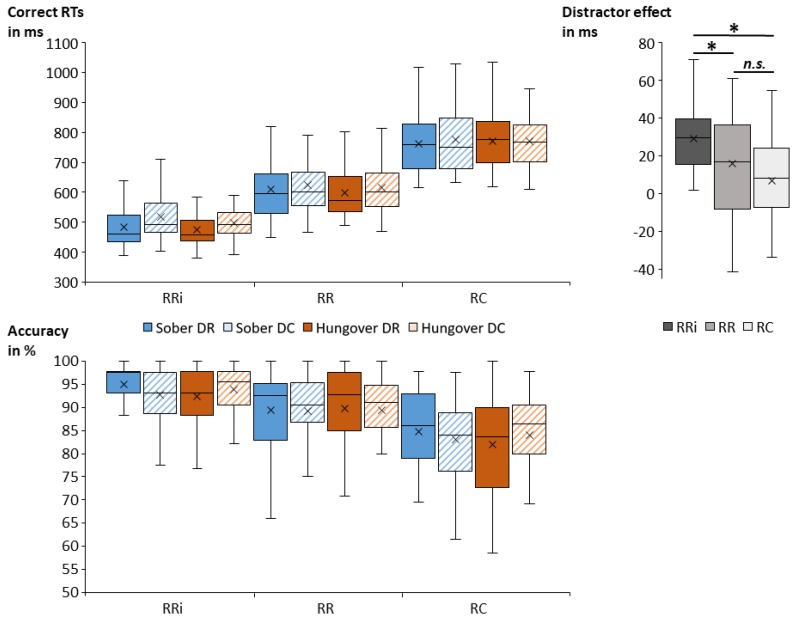
Box plots of the obtained mean correct probe response times (RTs in milliseconds, upper left graph) and mean probe accuracy (in percent, lower graph) for each combination of conditions. We observed significantly faster and more accurate responses in RRi than in RR and RC. We further observed significantly faster responses in DR than in DC. For probe response times, the size of the distractor effect (i.e., the difference between DC and DR, upper right graph) was significantly greater in RRi than in RR and RC, while it was not significantly different between RR and RC. While we found no significant main effects/overall differences between the sober and hangover session (i.e., irrespective of all other conditions), we found a significant interaction between status and distractor, which is depicted in Figure 5. Abbreviations: DR = distractor repetition; DC = distractor change; RRi = response repetition when the target stimulus was identical; RR = response repetition when the target stimulus differed; RC = response change; ms = milliseconds; * = *p* < 0.05; *n.s.* = non-significant.

The correct probe RTs and probe accuracy are illustrated in Figure 4.

The repeated-measures ANOVA for correct probe RTs (in case of correct prime response) revealed task-specific effects: There was a main effect of response (*F*_(2,68)_ = 374.42; *p* < 0.001; $\eta_{p}^{2}$ = 0.917). Post hoc comparisons revealed significant differences between all conditions, with the fastest responses in RRi trials (493 ms ± 9), followed by RR trials (613 ms ± 14), and RC trials (770 ms ± 13) (all *p* < 0.001). The main effect of distractor (*F*_(1,34)_ = 54.56; *p* < 0.001; $\eta_{p}^{2}$ = 0.616) showed faster responses in DR trials (617 ms ± 11) than in DC trials (634 ms ± 11). Furthermore, there was an interaction of response and distractor (*F*_(2,68)_ = 9.21; *p* = 0.001; $\eta_{p}^{2}$ = 0.213). Post hoc comparisons revealed that participants responded significantly faster in DR than in DC trials on RRi condition (*p* < 0.001; RRi-DR: 479 ms ± 9; RRi-DC: 508 ms ± 9), and on RR condition (*p* = 0.006; RR-DR: 605 ms ± 14; RR-DC: 621 ms ± 14), but not on RC condition (*p* = 0.254). We additionally computed the size of the distractor effect (DC minus DR) for each response condition. Post hoc comparisons revealed that the distractor effect in RRi trials (29 ms ± 3) was significantly greater than in RR trials (16 ms ± 5) (*p* = 0.023) and in RC trials (7 ms ± 4) (*p* ≤ 0.001). RR and RC trials did not significantly differ in the size of their distractor effect (*p* = 0.471). All main and interaction effects of status were non-significant (all *F* ≤ 1.16; all *p* ≥ 0.295).

**Figure 5:**
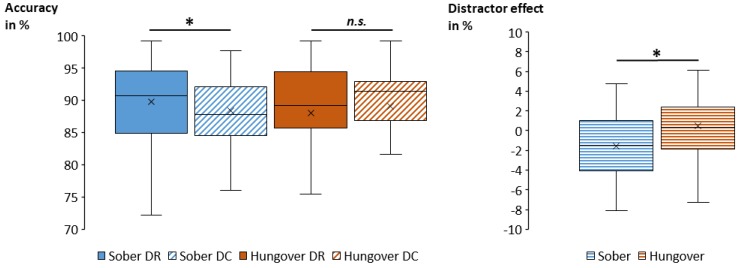
Box plots of the obtained mean probe accuracy (in percent) for the significant interaction of status and distractor are illustrated in the left graph. While the accuracy was significantly higher in DR trials (89.8 % ± 1.1) than in DC trials (88.4 % ± 1.1) when being sober, there was no such significant difference when being hungover. Consequently, the size of the distractor effect (the difference between DC and DR) was significantly smaller in the hangover assessment than in the sober assessment (right graph). Abbreviations: DR = distractor repetition; DC = distractor change; * = *p* < 0.05; *n.s.* = non-significant.

Regarding probe accuracy, the repeated-measures ANOVA showed a main effect of response (*F*_(2,68)_ = 79.07; *p* < 0.001; $\eta_{p}^{2}$ = 0.699). Post hoc comparisons revealed significant differences between all conditions, with the highest accuracy in RRi trials (93.5 % ± 0.7), followed by RR trials (89.4 % ± 1.0) and then by RC trials (83.5 % ± 1.2) (all *p* < 0.001). Importantly, an interaction of status and distractor was obtained (*F*_(1,34)_ = 5.02; *p* = 0.032; $\eta_{p}^{2}$ = 0.129), which is illustrated in Figure 5. Post hoc comparisons showed differences between distractor repetition and distractor change in the sober and hangover assessment. In the sober assessment, accuracy was significantly higher for DR trials (89.8 % ± 1.1) than for DC trials (88.4 % ± 1.1) (*p* = 0.027), while there was no significant distractor difference during the hangover assessment (*p* = 0.487). We additionally computed the size of the distractor effect (DC minus DR) for each status. A post hoc paired *t*-test showed that the size of the distractor effect was significantly smaller in the hangover assessment (|1.0| % ± 0.8) than in the sober assessment (|1.4| % ± 0.5) (*t*_(34)_ = |2.24|; *p* = 0.032). To investigate whether alcohol sensitivity or hangover severity modulated this effect, we correlated the overall ASQ score, the ASQ heavy score, and the overall hangover severity item by van Schrojenstein Lantman [13] with an average of all hungover DR trials, an average of all hungover DC trials, and the distractor effect size at hangover assessment as well as with the difference in distractor effect sizes between the sober and hangover assessment, and with the size of the hangover effect (SOB minus HANG) in DR and DC trials. None of these uncorrected correlations between ASQ scores, hangover severity, and performance parameters was significant (all *p* ≥ 0.064). All other main and interaction effects of the ANOVA were non-significant (all *F* ≤ 2.26; all *p* ≥ 0.118).

To summarize, we found that alcohol hangover affects the processing of response-irrelevant (distractor) information, but not the processing of response-relevant (target) information. The former is reflected by the interaction of status and distractor in probe accuracy, while the latter is reflected by non-significant hangover effects on response conditions in both correct probe RTs and probe accuracy.

## 4. Discussion

Distractibility is one of the major contributors of hangover-induced behavioral deficits in daily activities, like when driving a car [6,7,8,9,10,11,16]. As the basic mechanisms causing hangover-induced distractibility are still not very well understood, our study aimed to investigate the underlying mechanisms of distraction, as proposed by the TEC [20]. This theory postulates that stimulus-response (S-R) bindings are established in so-called event files. Given previous findings that sensory information seems to be accumulated more quickly when hungover [17], we hypothesized that S-R bindings should consequently be enhanced in the hungover state. We further hypothesized that alcohol hangover has different effects on the binding of response-relevant (target) information and the binding of response-irrelevant (distracting) information. To test these hypotheses, we used a paradigm by Moeller et al. [29] that allows to differentiate the effects of target-response binding and distractor-response binding. Each participant was tested once sober and once hungover after being intoxicated in a standardized experimental drinking setting the night before. Behavioral testing did not start until a BrAC of 0.00‰ was measured on both appointments.

Interestingly, alcohol hangover modulated distractor-response binding in the accuracy data, while target-response binding was not similarly affected: During the sober assessment, distractor repetition was advantageous in responding to the relevant target stimuli, and this effect was found regardless of target and response alterations (repetition vs. change). During the hungover assessment, this advantage of distractor repetition was no longer evident, as the distractor effect was significantly smaller. Of note, the sober distractor repetition effect can be explained by the TEC [20]: Whenever response-irrelevant distracting information is presented at the same time as response-relevant target information, both irrelevant and relevant stimulus features, as well as the response, are bound in event files [20,42]. Once the distracting information of the pre-existing event file reappears, the entire event file becomes (re)activated following a pattern-completion logic [20]. Changes in the configuration of presented stimuli and required response do however require readjustments of the current event file. As compared to distractor changes, distractor repetition should therefore require less extensive changes or adaptations in the reactivated event file. Hence, less cognitive resources should be needed in case of distractor repetition. This ultimately results in faster and more accurate responses. Even though the TEC may explain why and how distracting information modulates behavior in a sober state, this theory does not provide a straightforward explanation for the resulting pattern observed during alcohol hangover. Given that target-response binding was seemingly unaffected by hangover (as substantiated by the lack of interactions between response and status), it can be assumed that event files were still established during the hungover state. Within the framework of the TEC, the fact that the distractor effect became substantially smaller during hangover may be explained by reduced distractor processing and/or by enhanced target processing: Alcohol hangover may have affected the reactivation of the event file by recurrent distractor features. This would imply that the sensory processing of distracting information may be altered during hangover. Given that the accumulation of sensory information is likely more efficient in hangover state [17], we had initially expected distractor-response bindings to be strengthened. Based on Stock et al. [17], it was however not clear, whether all or only selected sensory information could be processed in a more efficient way. It is therefore also possible that relevant and irrelevant sensory information may be better discriminated during hangover. As a consequence, response-irrelevant distracting information might be processed to a lesser degree than during the sober state. By comparison, target information would then be processed more efficiently. In line with this, the typically higher activation of target information is assumed to have a greater impact on S-R bindings than the typically lower distractor information [43]. It could hence be speculated that the activation of the more dominant target information might be even higher during hangover due to its more efficient information accumulation [17]. Given that response and target repetition effects were however not enhanced during hangover, it seems more likely that the observed changes were primarily driven by decreased distractor processing, rather than by absolute increases in target processing. Interestingly, this may theoretically help to neutralize some of the cognitive side effects of alcohol hangover, rather than aggravate them. Further adding to this, the lack of target-associated effects matches the findings of a previous publication demonstrating that target-based S-R associations were not systematically modulated by acute alcohol intoxication [22].

Irrespective of hangover state, we found task-specific distractor-response binding effects in response times, which are in line with previous findings on the paradigm used in this study [30,44]: The event file-associated automatic response tendency induced by the distractor was advantageous in case of response repetition while the benefit of distractor repetition was no longer evident in case of response change. Additionally, the size of the distractor repetition effect was most pronounced in case of response and target repetition, while the effect was smaller in case of target change, regardless of the required response (repeated vs. changed). This result pattern may again be explained by the TEC: Whenever distractor repetition and response repetition coincide, the distractor reactivates an event file containing a response that is still correct in the current setting, thus resulting in faster responding. Whenever there is a response change in the presence of distractor repetition, the reactivated response no longer holds true for the current task requirement, resulting in a time-taking “unbinding” process of the event file [42,45]. Additionally, the reactivation of the event file is suggested to be stronger, the more stimulus and response features are repeated/activated [42,45]. That is, target and distractor repetition lead to a stronger reactivation and thus faster responding than distractor repetition alone, even when the response is the same in both cases (i.e., target change and target repetition). These sober findings are thus well in line with various studies investigating S-R binding [22,29,30,46].

Putting our results in a broader context, it should be noted that previous studies investigating distractor-induced cognitive and behavioral conflicts reported hangover-related impairments of selective attention when applying classical Stroop and Erikson flanker tasks [47,48]. Further detailing this, a previous study of our group showed that hangover enhanced the flanker effect only in case of (subliminally) increased conflict load, suggesting that the overall strain on cognitive control resources may play an important role for hangover effects on distractibility [18]. Yet, all these studies have in common that the applied tasks did not allow differentiation between response-relevant target and response-irrelevant distractor processing to the same degree as the current study. Furthermore, they put a stronger focus on conflict processing than on binding phenomena. While we only found selective impairments in distractor-response bindings, this does not exclude the possibility that other cognitive and attentional mechanisms, which were not assessed by our paradigm, may also be impaired and thus contribute to the hangover-related enhanced distractibility reported in other studies [14,47,48]. Therefore, further research is needed on other attentional mechanisms that could help explain increased distractibility in the hangover state. Furthermore, it would be conceivable to investigate these mechanisms in a naturalistic environment [49] and to investigate performance in real-life challenges like an actual exam or quantifiable work performance. This would help to gain insights into whether the consequences of alcohol hangover could be more severe in these situations and could help to derive better preventive measures.

### Limitations

The study sample was limited to males, as the ethics committee did not approve inducing such an intoxication in females. This is rather unfortunate, as other studies have reported females to experience more severe hangover symptoms than males [24,50], potentially because females tend to metabolite ethanol more slowly than males. In this light, females might experience greater hangover effects, which could translate to more severely impaired behavioral performance. Additionally, it has been suggested that the quality and incidences of experiencing alcohol hangover might change with age [51,52]. Based on these aspects, further studies including both sexes and adults of different age groups would provide a more complete picture. Furthermore, we did not collect data on drinking and detailed hangover history beyond the scope of the ASQ and AUDIT (which only covers the last 12 months). In doing so, we might have missed details on hangover and binge-drinking history, which could potentially have explained additional variance. In this context, it should however also be noted that as a result of the recruitment process, our study sample was quite homogenous with respect to drinking patterns. As our sample was likely to have shown comparatively minor differences in their hangover and/or binge-drinking history, we did not systematically investigate this. Studies that do not apply such inclusion criteria might however benefit from assessing more details on their sample’s drinking and hangover history.

## 5. Conclusions

In summary, we investigated whether and how alcohol hangover modulates S-R bindings, as this may explain why distractibility might be increased during hangover, resulting in various behavioral and performance difficulties. Specifically, we investigated whether hangover has different effects on target-response bindings vs. distractor-response bindings. While hangover did not affect target-response bindings, it decreased distractor-response bindings, resulting in the absence of distractor effects. These findings implicate that heightened distractibility during hangover is likely not due to increased distractor-response binding. Hence, other cognitive mechanisms relating to selective attention are more likely to underlie the adverse effects of hangover on driving skills and other daily activities. Therefore, more studies investigating the different aspects of distractibility and distractor processing are needed to conclusively understand how alcohol hangover may increase distractibility.

## Figures and Tables

**Table 1:** Sociodemographic, questionnaire, and alcohol-related data of the included sample. All values are reported as means ± standard error of the mean and (range). Please note that all relevant items were averaged for the ASQ_light_ score (alcohol-related experiences associated with lower doses). For the ASQ_heavy_ score (alcohol-related experiences associated with heavier doses), all relevant items except for “passing out” were averaged, as only a single participant had indicated to have ever passed out due to alcohol drinking.

Characteristic	Included Sample (*n* = 35)
Age in years	23.14 ± 0.47 (19–28)
Height in cm	181.89 ± 0.98 (170–195)
Weight in kg	76.87 ± 1.69 (63–105)
Cigarettes smoked per day	0.78 ± 0.37 (0–10)
Hours of sport per week	4.63 ± 0.56 (0–16)
BDI score	3.37 ± 0.69 (0–19)
AUDIT score	10.26 ± 0.58 (5–19)
ASQ total score	8.23 ± 0.40 (3.25–13.29)
ASQ_light_ score	5.16 ± 0.30 (1.63–9.25)
ASQ_heavy_ score	13.17 ± 0.78 (5.20–24.0)
Individual alcohol amount indicated in mL of brandy (36 vol %)	418.00 ± 5.81 (369–516)
Alcohol consumption duration in minutes	181.43 ± 4.29 (111–243)
BrAC 30 min after end of consumption	1.32 ± 0.03 (1.05–1.69)
BrAC 60 min after end of consumption	1.24 ± 0.02 (1.01–1.56)
BrAC 90 min after end of consumption	1.15 ± 0.02 (0.91–1.40)
BrAC 120 min after end of consumption	1.08 ± 0.03 (0.83–1.43)

BDI = Beck depression inventory; AUDIT = alcohol use disorders identification test; ASQ = alcohol sensitivity questionnaire; BrAC = breath alcohol concentration.

**Table 2:** Subjective ratings of sleep and hangover symptoms on both sessions. Hangover symptoms were rated on an 11-point Likert scale ranging from 0 points (no symptoms) to 10 points (extreme symptoms). Of note, participants were asked to truthfully rate the severity of each symptom on both sessions, irrespective of whether they had been drinking the night before the sober session. The mild symptom severity for the sober session and the resulting minimal variance of this rating may have contributed to the fact that nearly all hangover symptoms differed significantly between the sober and the hangover session (as was intended by the study). These comparisons were performed with uncorrected paired t-tests. *p*-Values are reported in the column “Difference.” All values are given as means ± standard error of the mean and (range).

Symptom	Sober	Hangover	Presence at Hangover Session	Difference
Hours of sleep in previous night	7.40 ± 0.15 (5.5–9)	5.54 ± 0.19 (4–8)	-	*p* < 0.001 **
Overall hangover severity	0 ± 0 (0–0)	3.63 ± 0.39 (0–10)	94.3%	*p* < 0.001 **
Thirst	0.91 ± 0.28 (0–7)	3.46 ± 0.37 (0–8)	91.4%	*p* < 0.001 **
Concentration problems	0.61 ± 0.18 (0–4)	3.11 ± 0.37 (0–8)	88.6%	*p* < 0.001 **
Tired	1.12 ± 0.21 (0–4)	4.57 ± 0.39 (1–10)	88.6%	*p* < 0.001 **
Sleepiness	1.00 ± 0.23 (0–4)	3.71 ± 0.42 (0–9)	85.7%	*p* < 0.001 **
Weakness	0.30 ± 0.11 (0–2)	2.31 ± 0.34 (0–10)	85.7%	*p* < 0.001 **
Headache	0.06 ± 0.04 (0–1)	2.69 ± 0.38 (0–8)	82.9%	*p* < 0.001 **
Clumsy	0.39 ± 0.15 (0–3)	1.97 ± 0.32 (0–6)	71.4%	*p* < 0.001 **
Dizziness	0.03 ± 0.03 (0–1)	1.69 ± 0.27 (0–6)	71.4%	*p* < 0.001 **
Sensitivity to light	0.24 ± 0.12 (0–3)	1.57 ± 0.32 (0–8)	60%	*p* < 0.001 **
Reduced appetite	0.21 ± 0.16 (0–5)	1.97 ± 0.41 (0–9)	57.1%	*p* = 0.001 **
Sweating	1.18 ± 0.33 (0–7)	1.09 ± 0.21 (0–4)	51.4%	*p* = 0.784
Nausea	0.03 ± 0.03 (0–1)	1.46 ± 0.35 (0–7)	48.6%	*p* < 0.001 **
Apathy	0 ± 0 (0–0)	0.89 ± 0.20 (0–4)	48.6%	*p* < 0.001 **
Shivering	0.36 ± 0.13 (0–3)	1.20 ± 0.29 (0–6)	45.7%	*p* = 0.010 *
Confusion	0.15 ± 0.08 (0–2)	0.91 ± 0.22 (0–5)	45.7%	*p* < 0.001 **
Heart pounding	0.30 ± 0.10 (0–2)	0.94 ± 0.23 (0–5)	42.9%	*p* = 0.012 *
Stomach pain	0.24 ± 0.16 (0–5)	1.06 ± 0.32 (0–7)	40%	*p* = 0.012 *
Anxiety	0.39 ± 0.14 (0–3)	0.94 ± 0.25 (0–5)	40%	*p* = 0.028 *
Regret	0.12 ± 0.12 (0–4)	0.83 ± 0.31 (0–10)	37.1%	*p* = 0.003 **
Sleeping problems	0.21 ± 0.14 (0–4)	0.91 ± 0.32 (0–8)	31.4%	*p* = 0.027 *
Depression	0.21 ± 0.16 (0–5)	0.66 ± 0.28 (0–9)	28.6%	*p* = 0.021 *
Vomiting	0.03 ± 0.03 (0–1)	0.60 ± 0.28 (0–9)	22.9%	*p* = 0.055
Heart racing	0.15 ± 0.08 (0–2)	0.57 ± 0.25 (0–6)	22.9%	*p* = 0.138

* *p* < 0.05, ** *p* < 0.01.

## Supplementary Materials

The following is available online at https://www.mdpi.com/2077-0383/9/3/778/s1.
