# Supplementary material for: Alcohol Hangover Differentially Modulates the Processing of Relevant and Irrelevant Information
Source: J Clin Med. 2020 Mar 12;9(3):778. doi: 10.3390/jcm9030778 (PMC7141188; doi:10.3390/jcm9030778)
Supplement: Supplementary file 1 [file jcm-09-00778-s001.pdf]

Supplementary Information

Article title:	**Alcohol hangover differentially modulates the processing of relevant and irrelevant information**
Journal name:	Journal of Clinical Medicine
Authors:	Antje Opitz, Christian Beste and Ann-Kathrin Stock
Corresponding author:	antje.opitz@ukdd.de

3. Results

3.3. Add-on analyses – alcohol hangover severity

To investigate whether cognitive performance differed between subjects experiencing light hangover symptoms and subjects with heavier hangover symptoms, we conducted a median split: All subjects who rated their hangover as "3" or less on the 0 to 10 point overall hangover severity item suggested by van Schrojenstein Lantman et al. [1] were classified as subjects with light hangover symptoms (n = 21). All other subjects were classified as subjects with heavy hangover symptoms (n = 14). We re-ran separate repeated-measures ANOVAs for correct probe response times (RTs) and probe accuracy with the additional between-subject factor of "*hangover severity group*" (light vs. heavy hangover symptoms). Regarding correct probe RTs, the repeated-measures ANOVA revealed a significant three-way interaction between hangover severity group, status and distractor ($F_{(1,33)} = 4.81$; $p = .035$; $\eta_p^2 = .127$). Yet, this effect did not survive post hoc testing as uncorrected independent t-tests showed no significant group differences for distractor repetition, distractor change, and the size of the distractor effect in both the sober and hungover state (all $t \leq |1.76|$; $p \geq .089$). All other interaction effects of hangover severity group were non-significant for the RT measure (all $F \leq 1.04$; $p \geq .350$). Regarding probe accuracy, the repeated-measures ANOVA showed no significant interaction between hangover severity group and any of the cognitive performance parameters (all $F \leq 1.47$; $p \geq .239$).

To investigate whether some of the single hangover symptoms were potentially more strongly associated with performance on the distractor-response binding paradigm than others, we ran uncorrected correlations between each hangover symptom as proposed by van Schrojenstein Lantman [1] and the behavioral hangover measures that had been shown to significantly differ from the sober pattern. The hungover performance in distractor repetition trials correlated negatively with feeling clumsy (r = -.387; p = .022) and with anxiety (r = -.344; p = .043). The hungover performance in distractor change trials correlated negatively with feeling clumsy (r = -.449; p = .007) and feeling weak (r = -.347; p = .041). The size of the hangover effect (sober minus hungover performance) in distractor change trials correlated positively with headache (r = .341; p = .045) and feeling weak (r = .362; p = .032). It should however be noted that none of these correlations would have survived Bonferroni corrections for multiple testing.

In summary, the data did not provide sufficiently reliable support for the assumption that hangover severity or specific hangover symptoms strongly modulated the observed overall hangover effects reported in the main manuscript. It should however also be noted that the sample was likely too small to detect medium, or even small-sized effects in this context.

4. Discussion

4.2. Impact of Hangover Symptoms

Interestingly, the most frequently reported hangover symptoms in our study (i.e., being thirsty, having concentration problems, being tired, and a feeling of sleepiness), are in line with the most frequently reported symptoms in another study by van Schrojenstein Lantman [2]. Likewise, these four

hangover symptoms were the most severe experienced symptoms in terms of the subjective rating. However, none of them were related to cognitive performance in the distractor-response binding paradigm. Instead, subjects who reported feeling more clumsy, weak, or anxious seemed to perform less accurately in the hangover state, although these symptoms seemed to be rather mild complaints according to their severity scoring. This is partly in line with another study [2] where ratings of feeling clumsy and weak were of comparable frequency and severity and had a rather moderate impact on cognitive functioning. Opposing our findings, that study reported that symptoms which are usually low in frequency and severity, like anxiety, had only little impact on cognition [2]. Additionally, symptoms high in frequency and severity, like concentration problems, did also correspond well with high impact on cognitive functioning [2], which in our data seemed to hold only true for headache. Nevertheless, these interpretations should be treated with caution as our correlational analyses were only add-on and not sufficiently reliable as we did not apply Bonferroni corrections and the sample size was likely to small.

References

1. van Schrojenstein Lantman, M.; van de Loo, A.; Mackus, M.; Verster, J. Development of a Definition for the Alcohol Hangover: Consumer Descriptions and Expert Consensus. *Curr. Drug Abuse Rev.* **2017**, *9*, 148–154.
2. van Schrojenstein Lantman, M.; Mackus, M.; van de Loo, A.J.A.E.; Verster, J.C. The impact of alcohol hangover symptoms on cognitive and physical functioning, and mood. *Hum. Psychopharmacol. Clin. Exp.* **2017**, *32*, e2623.
